# Red blood cell distribution width as a predictor of mortality among patients regularly visiting the nephrology outpatient clinic

**DOI:** 10.1038/s41598-021-03530-2

**Published:** 2021-12-21

**Authors:** Kyung Don Yoo, Hyung Jung Oh, Sehoon Park, Min Woo Kang, Yong Chul Kim, Jae Yoon Park, Jeonghwan Lee, Jong Soo Lee, Dong Ki Kim, Chun Soo Lim, Yon Su Kim, Jung Pyo Lee, Korean Association for the study of Renal Anemia and artificial Intelligence (KARAI), Dong Ki Kim, Chun Soo Lim, Jung Pyo Lee

**Affiliations:** 1 Department of Internal Medicine, Ulsan University Hospital, University of Ulsan College of Medicine, Ulsan, Korea; 2 Department of Nephrology, Sheikh Khalifa Specialty Hospital, Ras al Khaimah, United Arab Emirates; 3 Department of Internal Medicine, Seoul National University Hospital, Seoul, Korea; 4 Department of Internal Medicine, Dongguk University College of Medicine, Dongguk University Ilsan Hospital, Seoul, Korea; 5 Department of Internal Medicine, Seoul National University Boramae Medical Center, Seoul, Korea; 6 Department of Internal Medicine, Seoul National University College of Medicine, Seoul, Korea; 7 Kidney Research Institute, Seoul National University College of Medicine, Seoul, Korea

**Keywords:** Nephrology, Haematological diseases

## Abstract

The association between increased red blood cell distribution width (RDW) and mortality among patients treated on an outpatient basis in the nephrology outpatient clinic is unclear. Therefore, our study aimed to investigate the association between baseline and time-averaged RDW and mortality risk in patients treated in our nephrology outpatient clinic. Our multi-center retrospective analysis was based on data of 16,417 outpatient nephrology patients with available baseline renal function and RWD values. The median baseline RDW was 13.0% (range, 10.0–32.1%). The high-RDW group was defined as the top quartile (≥ 13.8%, *n* = 4302). The crude mortality rate was 15.0% (*n* = 1806) at a median follow-up of 127.5 months. From the results of the multivariate Cox proportional hazards regression model adjusted for covariates, including eGFR, hemoglobin, and factors of anemia treatment, patients with a high time-averaged RDW had increased mortality risk (adjusted hazard ratio, 1.505; 95% confidence interval, 1.326–1.708; *P* < 0.001), irrespective of sex, presence of anemia, and chronic kidney disease, except in individuals aged < 45 years. Thus, increased baseline and time-averaged RDW were significantly associated with increased mortality in patients aged > 45 years treated on an outpatient basis in the nephrology clinic.

## Introduction

The red blood cell distribution width (RDW) reflects the degree of heterogeneity in red blood cell (RBC) volume and is used as a parameter to measure variations in the erythrocyte width reported by hemocytometers^1^ and to differentiate various hematologic diseases. Several reports have recently indicated that baseline RDW and/or change in RDW is significantly associated with the incidence of not only cardiovascular events but also acute clinical adverse effects in critically ill patients^2^. Several studies have revealed a relationship between RDW and renal diseases. Lippi et al. investigated the relationship between RDW and glomerular filtration rate (GFR) in a large Italian cohort of unselected outpatients^3^. Other studies reported that higher RDW at baseline was significantly associated with higher mortality rate in patients with advanced chronic kidney disease (CKD)^4^ or acute kidney injury^5^, as well as in those undergoing hemodialysis^6^ or peritoneal dialysis^7^. While the mechanism underlying the relationship has not been clearly explained, erythropoietin production, chronic inflammation, and/or oxidative stress might be involved in the association. Nonetheless, most of these studies were conducted on patients with advanced CKD, including those on dialysis who required frequent administration of erythropoietin-stimulating agents and blood transfusion, suggesting that they were more likely to have anemia^8^.

However, to the best of our knowledge, no study has evaluated the effectiveness of RDW in predicting mortality in this clinical population. Therefore, our aim in this study was to investigate the association between baseline and time-averaged RDW and mortality risk in patients treated in our nephrology outpatient clinic. This was feasible as blood tests, such as complete blood count (CBC) with RDW, are usually performed in all patients treated in our nephrology outpatient clinic and the cost for such tests is low.

## Methods

### Study participants

**Figure 1:**
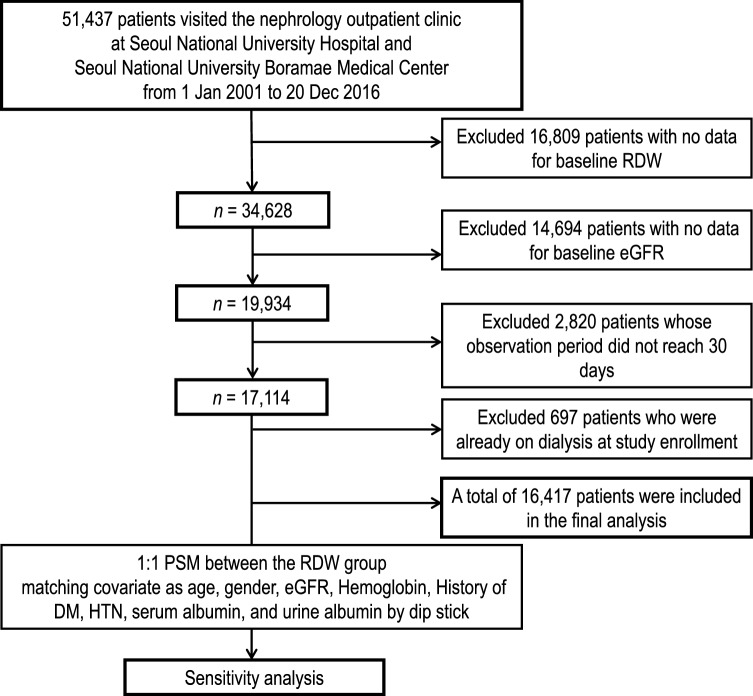
Study flow chart.

A retrospective observational cohort study was conducted on patients tracked from the outpatient nephrology clinic of two tertiary hospitals in South Korea (Seoul National University Hospital and Seoul National University Boramae Medical Center). A total of 51,437 patients were screened from January 2001 to December 2016. Among those screened, 16,809 patients who had no baseline RDW values, 14,694 patients who had no baseline estimated GFR (eGFR) values, 2820 patients who died within 30 days of the initial outpatient clinic visit, and 697 patients who underwent dialysis before the index date of study enrollment were excluded. Consequently, a total of 16,417 patients were included in the final analysis (Fig. 1).

### Data collection

Data on baseline characteristics, including demographic information and preexisting chronic comorbidities, such as hypertension and diabetes mellitus (DM), were collected. Age, sex, body mass index (kg/m^2^), systolic and diastolic blood pressures, data related to other comorbidities (using ICD-10 diagnosis codes), and medication information were recorded. Serum creatinine and albumin concentrations were measured, and the urinary albumin grade was determined by urine analysis. The eGFR was calculated using the Modification of Diet in Renal Disease (MDRD) study equation^9^.

Laboratory findings, including a complete blood count (CBC) panel with differential counts, were acquired. RDW was measured by a hematology analyzer (Sysmex XE-2100) and was recorded using the same CBC panel test each time that patients visited the outpatient clinic. In this study, the RDW values were reported as coefficients of variation (percentages) of the RBC volume, and the normal reference range of RDW was 13.5% ± 2.5%. Patients were stratified into two groups according to their RDW value: a high-RDW group, which comprised patients in the highest RDW quartile (RDW ≥ 13.8%), and a normal-RDW group, which comprised all other patients. Furthermore, the effects of time-dependent changes in RDW on mortality and the incidence of end-stage renal disease (ESRD) were investigated using time-averaged RDW values. Laboratory results from the time of study enrollment (baseline) and those nearest to the 3-, 6-, 9-, and 12-month follow-up periods were retrieved to calculate the time-averaged RDW. Furthermore, laboratory results for serum creatinine level, eGFR calculated using the MDRD equation, and hemoglobin level were obtained at each time point for the time-averaged values as covariates. Additionally, the association between increased RDW and clinical outcomes was analyzed in each subgroup classified according to the presence or absence of anemia. Data on the use of erythropoietin-stimulating agents and RBC transfusion, which are the two major RDW-associated treatments for anemia, were also collected. The World Health Organization’s definition of anemia was adopted for this study^10^.

### Ethical issues

All clinical studies were conducted in accordance with the guidelines of the 2013 Declaration of Helsinki. The present study was approved by the Seoul National University Hospital and Seoul National University Boramae Medical Center ethics committee/institutional review board (IRB no. 20200302/30-2020-17/033) and was exempted from informed consent requirements owing to its retrospective design.

### Clinical outcomes

The primary outcomes were all-cause mortality and the rate of patients requiring maintenance dialysis. With respect to patient mortality, information was obtained from the National Statistics Office^11^.

### Statistical analysis

Demographic data were compared using the chi-square test and Student’s *t*-test for categorical and continuous variables, respectively. Categorical variables were presented as the percentage of all patients, whereas continuous variables were expressed as mean ± standard deviation. A multivariate Cox proportional hazards model was used to examine the hazard ratio (HR) of the high-RDW group and 1-unit increase in RDW as a continuous variable with 95% confidence interval (CI) for the primary outcome. Kaplan–Meier curves were plotted for the groups in the survival analysis, and multivariate Cox proportional hazards model curves were also presented with baseline RDW using HR and adjusted HR. Furthermore, the effects of time-dependent changes in RDW on mortality and the incidence of ESRD were investigated using time-averaged RDW. The association of increased RDW with mortality and ESRD was analyzed in each subgroup classified according to age (≥ 60 or < 60 years), sex (male or female), presence or absence of anemia, and current renal function (eGFR ≥ 60 or < 60 mL/min/1.73 m^2^). Multivariate Cox analyses were performed in each group to reveal the association of baseline RDW or time-averaged RDW with mortality and ESRD. The covariates adjusted for were age, sex, eGFR, hemoglobin level, history of DM, history of hypertension, serum albumin level, urinary albumin grade, history of RBC transfusion, and use of erythropoietin-stimulating agents for each subgroup (excluding own). The propensity score matching (PSM) and standardized differences were used to compare the baseline characteristics of the two groups for sensitivity analysis^12^. There were some differences in the observed baseline characteristics between the two groups, and those differences could result in biased estimates of the RDW effect^13^. Propensity scoring has been used to reduce bias in observational studies by balancing the covariates between groups^12,13^. In this study, the nearest neighbor matching methods and a 1:1 matching algorithm without replacement were used to determine a match for each individual in the RDW group. All statistical analyses were conducted using R version 3.5.0 (Comprehensive R Archive Network; http://cran.r-project.org, MatchIt package for PSM^14^) and SPSS version 22.0 (IBM Corp., Armonk, NY, USA). In all analyses, *P* < 0.05 was considered to indicate statistical significance.

### Ethics approval and consent to participate

Our study was conducted in accordance with the guidelines of the 2013 Declaration of Helsinki and was approved by the Seoul National University Hospital and Seoul National University Boramae Medical Center (no. 20200302/30-2020-17/033). The requirement for informed consent was waived owing to the retrospective design of the study.

## Results

### Baseline characteristics and demographic data

**Figure 2:**
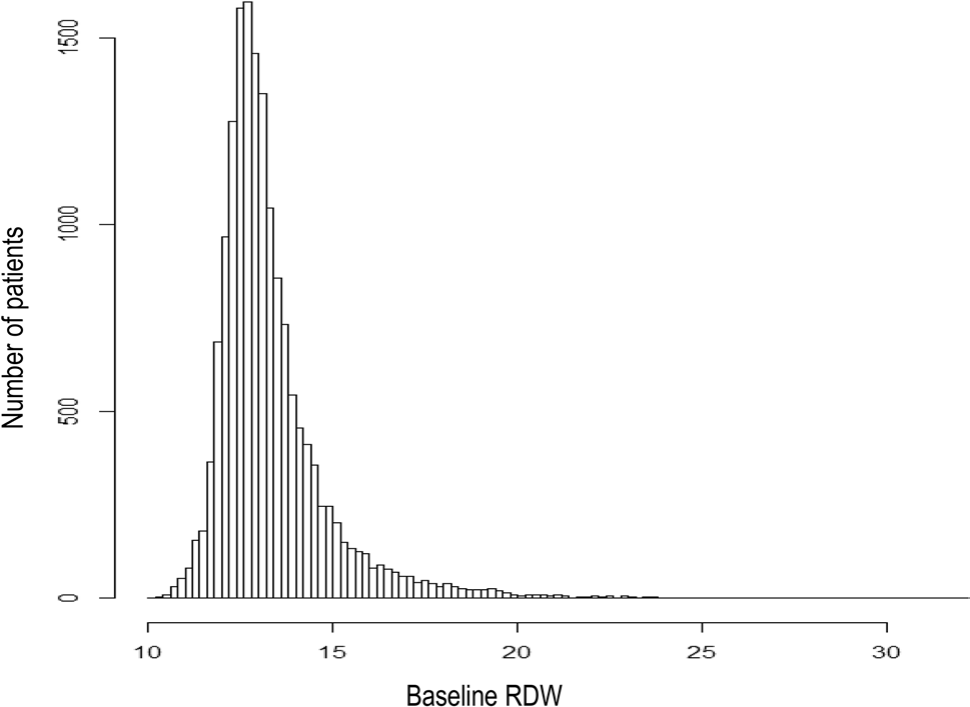
Distribution of red blood cell distribution width (RDW) for the study cohort.

Our analysis was based on data from 16,417 patients. The baseline characteristics and demographic data of the study group are summarized in Table 1 & Table S2. The distribution of baseline RDW values is presented in Fig. 2, and the distribution as a function of the presence/absence of anemia is shown in Supplemental Fig. S1. Of the 16,417 patients included in our analysis, 4302 (26.2%) were in the highest RDW quartile (RDW ≥ 13.8%). The high-RDW group had a higher number of older and male patients, as well as a higher proportion of patients with advanced CKD and comorbidities, such as hypertension and DM, than the normal-RDW group. The mean hemoglobin level was lower in the high-RDW group (10.8 g/dL) than in the normal-RDW group (13.0 g/dL). The crude mortality and ESRD incidence rates were higher in the high-RDW group than in the normal-RDW group (mortality: 15.0% vs. 9.6%, *P* < 0.001; ESRD incidence: 13.5% vs. 11.2%, *P* < 0.001; Table 1).

**Table 1:** Baseline characteristics of participants by RDW group.

	Normal-RDW group^a^	High-RDW group^a^	*P*
Count	12,115 (73.8%)	4302 (26.2%)	
Age (years)	55.0 [41.0, 66.1]	57.0 [43.7, 68.4]	< 0.001
**Sex**			0.042
Female	6224 (51.4%)	2132 (49.6%)	
Male	5891 (48.6%)	2170 (50.4%)	
Body mass index (kg/m^2^)	23.6 [21.2, 26.1]	23.1 [20.6, 25.5]	< 0.001
Systolic blood pressure (mm Hg)	130.0 [117.0, 146.0]	130.0 [118.0, 147.0]	0.438
Diastolic blood pressure (mm Hg)	79.0 [70.0, 88.0]	79.0 [70.0, 88.0]	0.839
Hemoglobin (g/dL)	13.0 [11.6, 14.4]	10.8 [9.4, 12.4]	< 0.001
Serum creatinine (mg/dL)	1.1 [0.9, 1.7]	1.4 [1.0, 2.2]	< 0.001
Estimated glomerular filtration rate (mL/min/1.73 m^2^)^b^	61.0 [38.1, 89.2]	46.4 [27.1, 72.8]	< 0.001
**CKD grade**			< 0.001
CKD grade 1	2931 (24.2%)	651 (15.1%)	
CKD grade 2	3266 (27.0%)	888 (20.6%)	
CKD grade 3	3866 (31.9%)	1537 (35.7%)	
CKD grade 4	1254 (10.4%)	610 (14.2%)	
CKD grade 5	798 (6.6%)	616 (14.3%)	
**Urinary albumin grade by urine analysis**			< 0.001
Negative	6233 (55.1%)	1872 (47.1%)	
1 +	1016 (9.0%)	512 (12.9%)	
2 +	1921 (17.0%)	808 (20.3%)	
3 +	1782 (15.8%)	655 (16.5%)	
4 +	360 (3.2%)	126 (3.2%)	
Serum albumin	4.2 [3.9, 4.4]	3.9 [3.5, 4.2]	< 0.001
Prevalence of anemia	3980 (37.2%)	2899 (75.8%)	< 0.001
History of hypertension	3782 (31.2%)	1554 (36.1%)	< 0.001
History of diabetes mellitus	2512 (20.7%)	965 (22.4%)	0.019
Crude mortality rate	1160 (9.6%)	646 (15.0%)	< 0.001
Crude end-stage renal disease incidence rate	1354 (11.2%)	579 (13.5%)	< 0.001

^a^The threshold between normal and high RDW was defined as the upper level of the third quartile (RDW ≥ 13.8%).

^b^Calculated using the Modification of Diet in Renal Disease equation^9^.

Values are presented as *n* (%) or as mean [interquartile range].

*CKD* chronic kidney disease, *RDW* red blood cell distribution width.

### Association between RDW and mortality

**Figure 3:**
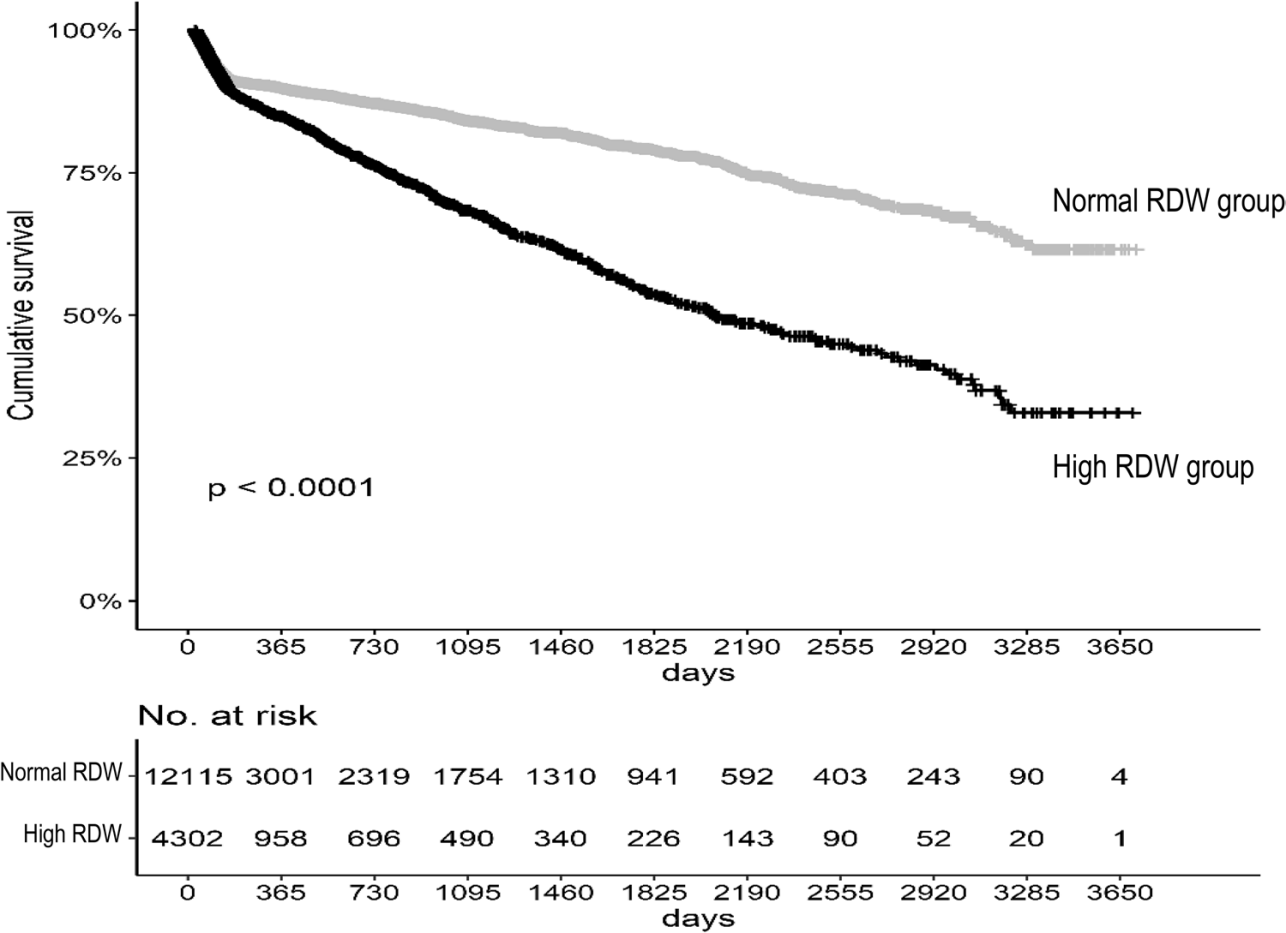
Survival analysis of all-cause mortality among the baseline red blood cell distribution width (RDW) groups. The threshold between normal and high RDW was defined as the upper level of the third quartile (RDW ≥ 13.8%).

**Figure 4:**
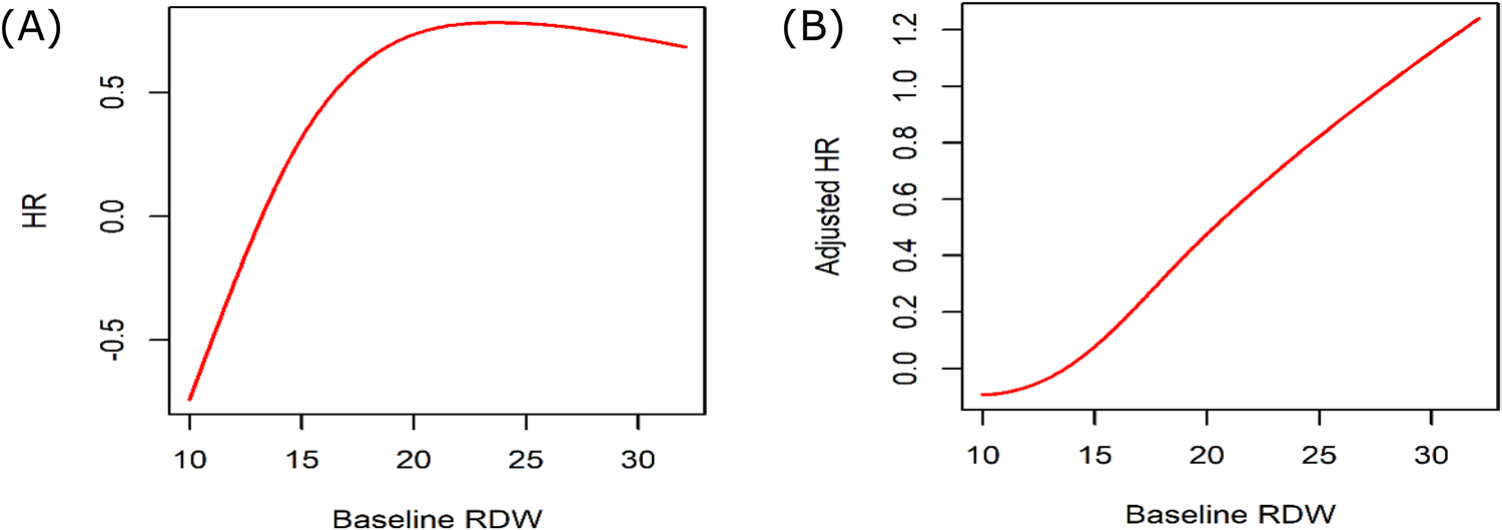
Nonlinear association between red blood cell distribution width (RDW) values and mortality risk. Adjustments were made for age, sex, estimated glomerular filtration rate, and hemoglobin level. (**A**) Univariate analysis. (**B**) Multivariate analysis.

At a median follow-up of 127.5 months (10.63 years), the overall crude mortality rate and crude ESRD incidence rate were 11.0% and 11.7%, respectively, with the mortality and ESRD rate increasing steadily over time. The Kaplan–Meier survival analysis revealed a higher mortality risk in the high-RDW group than in the normal-RWD group (log-rank *P* < 0.0001; Fig. 3). Similarly, compared to the normal time-averaged RDW group, the high time-averaged RDW group showed an increased cumulative incidence of mortality (Supplementary Fig. S2). This increase was confirmed with the univariate Cox proportional hazards regression model findings, which indicated a significantly higher mortality risk for the high-RDW group than for the normal time-averaged RDW group (hazard ratio (HR), 1.741; 95% confidence interval (CI), 1.581–1.917; *P* < 0.001). The higher mortality risk for the high-RDW group than for the normal-RDW group remained significant even after adjustment for covariates, which included information on treatment for anemia (HR, 1.325; 95% CI, 1.167–1.504; *P* < 0.001; model C). When the effect of RDW was investigated as a continuous variable for mortality risk using a multivariate Cox regression analysis, a 1-unit increase in baseline RDW was associated with 7.4% increase in all-cause mortality (Table 2). Assessment of the nonlinear association between RDW and mortality risk is presented in Fig. 4. The association between the time-averaged RDW and mortality risk is reported in Table 2. The HR for mortality was significantly higher in the high time-averaged RDW group than for the normal time-averaged RDW group (HR, 1.505; 95% CI, 1.326–1.708; *P* < 0.001). The multivariate Cox regression analysis revealed that 1-unit increase in time-averaged RDW was associated with 15.8% increase in the all-cause mortality rate (Table 2; Supplementary Fig. S3).

**Table 2:** Cox regression analysis of the association between baseline RDW and all-cause mortality.

	Univariate		Model A		Model B		Model C	
	HR (95% CI)	*P*	Adjusted HR^a^ (95% CI)	*P*	Adjusted HR^b^ (95% CI)	*P*	Adjusted HR^c^ (95% CI)	*P*
High-RDW group (vs. normal-RDW group)	1.741 (1.581–1.917)	< 0.001	1.290 (1.152–1.445)	< 0.001	1.335 (1.177–1.514)	< 0.001	1.325 (1.167–1.504)	< 0.001
Baseline RDW (as a continuous variable, per 1-unit increase)	1.134 (1.108–1.160)	< 0.001	1.068 (1.035–1.101)	< 0.001	1.077 (1.042–1.077)	< 0.001	1.074 (1.038–1.038)	< 0.001
High time-averaged RDW group (vs. normal time-averaged group)^d^	1.942 (1.765–2.138)	< 0.001	1.365 (1.226–1.520)	< 0.001	1.499 (1.324–1.698)	< 0.001	1.505 (1.326–1.708)	< 0.001
Time-averaged RDW (as a continuous variable, per 1-unit increase)^d^	1.246 (1.213–1.280)	< 0.001	1.135 (1.096–1.176)	< 0.001	1.158 (1.115–1.203)	< 0.001	1.158 (1.114–1.205)	< 0.001

^a^Model A was adjusted for age, sex, estimated glomerular filtration rate, and baseline hemoglobin level.

^b^Model B was adjusted for all covariates in model A plus history of diabetes mellitus, history of hypertension, serum albumin level, and urinary albumin grade.

^c^Model C was adjusted for all covariates in model B plus history of red blood cell transfusion and use of erythropoietin-stimulating agents.

^d^When analysis was performed with time-averaged RDW values, the time-averaged hemoglobin levels and time-averaged estimated glomerular filtration rate were included in the multivariate Cox regression model.

*CI* confidence interval, *HR* hazard ratio, *RDW* red blood cell distribution width.

### Subgroup analysis

**Figure 5:**
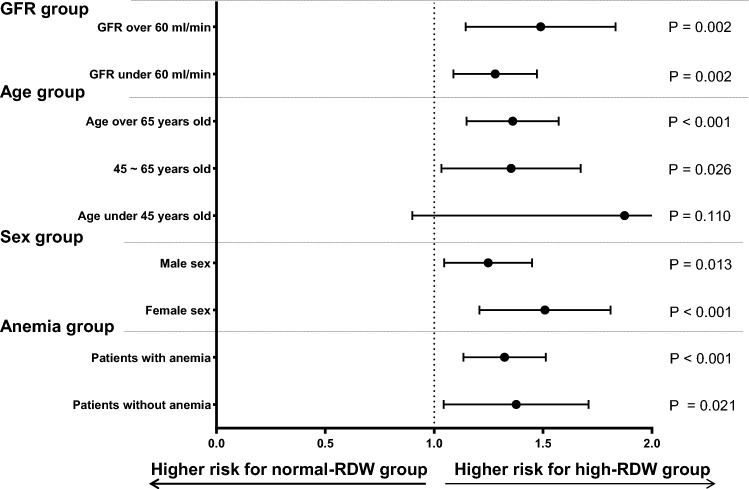
Multivariate Cox regression analysis of high-RDW vs. normal-RDW mortality by subgroup. The adjusted covariates were age, sex, estimated glomerular filtration rate, hemoglobin level, history of diabetes mellitus, history of hypertension, serum albumin level, urinary albumin grade, history of red blood cell transfusion, and use of erythropoietin-stimulating agents. *GFR* glomerular filtration rate, *RDW* red blood cell distribution width.

To identify the effects of baseline or time-averaged RDW on mortality risk under diverse conditions, we stratified patients into groups according to age (≥ 65, 45–65, and < 45 years), sex (male or female), presence or absence of anemia, and renal function (eGFR ≥ 60 or < 60 mL/min/1.73 m^2^). The risk of mortality was significantly higher in each subgroup of the high-RDW group and the high time-averaged RDW group than in the equivalent subgroups of the normal-RDW and normal time-averaged RDW groups, irrespective of sex, presence of anemia, or renal function (Fig. 5, Supplemental Fig. S4). However, there was no significant association between the RDW and all-cause mortality for patients aged < 45 years after adjustment for confounding factors (high-RDW group: HR, 1.160; 95% CI, 0.900–2.849; *P* = 0.110; high time-averaged RDW group: HR, 1.265; 95% CI, 0.700–2.288; *P* = 0.436) (Fig. 5). The effect of RDW on mortality risk was comparable for the different RDW quartiles (Supplemental Table S1, Supplemental Figs. S5 and S6).

### Sensitivity analysis

We performed additional sensitivity analyses after propensity score matching (PSM) to overcome any partial bias. We effectively corrected for the analysis of subgroups and adjusted the covariates to avoid a partial bias that could still affect the interpretation of results. Figure S7 and Table S3 show the baseline characteristics after PSM. Table S3 showed there were no significant differences in age, sex, eGFR, hemoglobin, and history of DM and hypertension between the two groups after PSM. At the final analysis, when we performed the multivariate Cox analysis, the high-RDW group was still significantly associated with increased mortality rate in the final model C in the matched population (high-RDW group: HR, 1.329; 95% CI, 1.155–1.529; *P* < 0.001; high time-averaged RDW group: HR, 1.530; 95% CI, 1.326–1.765; *P* < 0.001) (Table S4).

## Discussion

In this study, we investigated the association between RDW and mortality among patients treated on an outpatient basis in our nephrology clinic. Higher RDW values at baseline were more significantly related to higher mortality rates than lower RDW values. Moreover, this association was identified even with time-averaged RDW, irrespective of age > 45 years, sex, or eGFR at baseline. However, no significant association between RDW and mortality was observed among those aged < 45 years.

Several studies have revealed a relationship between RDW and adverse clinical outcomes^15–18^. Most of their results are consistent with ours. The mechanism underlying the association between RDW and mortality is poorly understood. Nonetheless, several plausible explanations concerning the underlying biological mechanism have been proposed. Systemic factors that alter erythrocyte homeostasis, such as inflammation, malnutrition, and oxidative stress, are considered to be involved in the mechanism. Lippi et al.^3^ showed a positive correlation between RDW and inflammatory markers, such as erythrocyte sedimentation rate and high-sensitivity C-reactive protein, in a large cohort of unselected outpatients. Moreover, Zurauskaite et al. reported a strong correlation between RDW and malnutrition^19^. Several studies found an association of RDW with oxidative stress and reported increased anisocytosis resulting from disruption of erythropoiesis, blood cell membrane deformity, and alteration in circulating erythrocyte half-life under oxidative stress, ultimately leading to increased RDW^15,20,21^.

As noted by Yoon et al.^22^, a progressive increase in RDW can independently predict mortality and cardiovascular events in patients with ESRD. Previous studies showed the correlation between low GFR, microalbuminuria, and elevated RDW values^3,23,24^. We speculate that renal dysfunction may be linked to several other risk factors that can increase RDW values, such as malnutrition, anemia, inflammation, and oxidative stress. Renal dysfunction may be a comprehensive manifestation of various pathophysiological processes. Nevertheless, to the best of our knowledge, no study has investigated the association between RDW and mortality in patients visiting nephrology outpatient clinics, even though blood tests are routinely performed whenever patients visit these clinics.

Patel et al. found an association of RDW with mortality risk in middle-aged and older adults and showed that RDW was a significant predictor of all-cause mortality even in non-anemic participants (baseline hemoglobin > 13.0 g/dL in women and > 14.0 g/dL in men)^15^. They concluded that RDW is a widely available indicator that can predict mortality among adults aged ≥ 45 years in the general population. All our results were consistent with these findings. However, we found no significant association of RDW with mortality among adults aged < 45 years. It is difficult to explain the non-significant association between RDW and mortality in younger participants. However, there were large differences in crude mortality incidence among the groups. The mortality rate was 2.0% (97/4780) in individuals aged < 45 years, 7.9% (540/6843) in individuals between 45 and 60 years of age, and 24.4% (1169/4794) in individuals > 60 years of age during the follow-up period (429 ± 697 days) (*P* < 0.001). Too-low mortality rates could lead to no significant relationship between RDW and mortality. Moreover, because more participants had normal-ranged RDW in the younger group as compared to the other groups, we surmise that there might be no significant association among them. Therefore, caution is advised when interpreting the results for younger adults aged < 45 years. Alcaino et al. reported RDW as a biomarker in older adults; several alterations, such as an increased number of cardiovascular diseases, cancer, and poor nutritional status, are usually more common in older adults, and they hypothesized that RDW is a “master” blood biomarker reflecting the general health status in the elderly^25^. To our knowledge, there has been no study on the effect of RDW on mortality among adults < 45 years. The relationship between an increase in RDW and mortality in younger adults may be different from that in older adults, and the current study showed no significant association between RDW and mortality in adults < 45 years. In the future, a larger study including a younger health population will be needed to reveal the effect of RDW on mortality.

The present study has several limitations that should be acknowledged in the interpretation of our results. First, this study had a retrospective cohort design. While we collected full data for patients visiting the nephrology department, selection bias could still have occurred throughout the study. Second, the data were obtained from only two hospitals in South Korea; hence, caution is needed in interpreting the results due to restricted ethnicity and location. Third, a total of 2820 patients who died within 30 days of the start of study enrollment were excluded. We attempted to investigate the association between increased RDW and mortality among participants visiting the nephrology outpatient clinic, implying that most of them were hemodynamically stable. Those patients who died within 30 days of study enrollment were considered to be hemodynamically unstable at the enrollment time point. Fourth, 697 patients on chronic dialysis were additionally excluded because we wished to examine the effects of RDW on mortality among participants requiring regular visits to the nephrology outpatient clinic except for those on dialysis. Moreover, Yoon et al.^22^ already reported an association between RDW and mortality in patients with ESRD. In addition to the above-mentioned limitations, patients who received transfusions and erythropoietin-stimulating agents were also excluded before enrollment in this study, and we could thus exclude the unexpected effects of transfusion and erythropoietin-stimulating agents on increased RDW.

In conclusion, this study showed that increased baseline and time-averaged RDW values were significantly associated with increased mortality in patients visiting nephrology outpatient clinics, especially among those > 45 years of age.

## Supplementary Information

Supplementary Information.

Supplementary Figures.

## Data Availability

The datasets used and/or analyzed during the current study are available from the corresponding author on reasonable request.
