# Supplementary material for: Red blood cell distribution width as a predictor of mortality among patients regularly visiting the nephrology outpatient clinic
Source: Sci Rep. 2021 Dec 21;11:24310. doi: 10.1038/s41598-021-03530-2 (PMC8692533; doi:10.1038/s41598-021-03530-2)
Supplement: Supplementary file 1 — Supplementary Information. [file 41598_2021_3530_MOESM1_ESM.doc]

**Red blood cell distribution width as a predictor of mortality among patients regularly visiting the nephrology outpatient clinic**

Kyung Don Yoo^1,8^, Hyung Jung Oh^2,8^, Sehoon Park^3^, Min Woo Kang^3^, Yong Chul Kim^3^, Jae Yoon Park^4^, Jeonghwan Lee^5^, Jong Soo Lee^1^, Dong Ki Kim^3,6,7^, Chun Soo Lim^5,6,7^, Yon Su Kim^3,6,7^, Jung Pyo Lee^5,6,7^, on behalf of the Korean Association for the study of Renal Anemia and artificial Intelligence (KARAI)^*^

^1^Department of Internal Medicine, Ulsan University Hospital, University of Ulsan College of Medicine, Ulsan, Korea

^2^Department of Nephrology, Sheikh Khalifa Specialty Hospital, United Arab Emirates

^3^Department of Internal Medicine, Seoul National University Hospital, Seoul, Korea

^4^Department of Internal Medicine, Dongguk University College of Medicine, Dongguk University Ilsan Hospital, Seoul, Korea

^5^Department of Internal Medicine, Seoul National University Boramae Medical Center, Seoul, Korea

^6^Department of Internal Medicine, Seoul National University College of Medicine, Seoul, Korea

^7^Kidney Research Institute, Seoul National University College of Medicine, Seoul, Korea

^8^KDY and HJO contributed equally to this research.

*A list of authors and their affiliations appears at the end of the paper.

**Corresponding author:**

**Jung Pyo Lee**

Email: [nephrolee@gmail.com](mailto:nephrolee@gmail.com)

**Supplementary Figure Legends**

**Figure S1. Distribution of baseline red blood cell distribution width (RDW) according to the presence/absence of anemia.** (A) Anemia group. (B) Non-anemia group.

**Figure S2. Survival analysis of all-cause mortality according to the time-averaged red blood cell distribution width (RDW) groups**.

**Figure S3. Analysis of the non-linear association between the time-averaged red blood cell distribution width (RDW) values and mortality risk among patients adjusted for age, sex, time-averaged estimated glomerular filtration rate, and time-averaged hemoglobin level.** When plotting the association between time-averaged RDW and adjusted hazard ratio (HR) for mortality risk, the time-averaged estimated glomerular filtration rate and time-averaged hemoglobin levels were included in the multivariate Cox regression model.

**Figure S4. Multivariate Cox regression analysis of *the* *high* time-averaged RDW group compared to *the normal* group *on mortality* in each subgroup.**

The covariates adjusted for were age, sex, time-averaged estimated glomerular filtration rate, time-averaged hemoglobin level, history of diabetes mellitus, history of hypertension, serum albumin level, urinary albumin grade, history of red blood cell transfusion, and use of erythropoietin-stimulating agents. Abbreviations: GFR, glomerular filtration rate; RDW, red blood cell distribution width.

**Figure S5. Patient mortality according to the red blood cell distribution width (RDW) quartiles measured at baseline.**

**Figure S6. Patient mortality according to the time-averaged red blood cell distribution width (RDW) quartiles.**

**Figure S7. Standardized difference (SD) before and after propensity-score matching**

(A) Change of SD value for before and after propensity-score matching by variables

(B) Distribution of SD for before and after propensity-score matching

**Supplementary Tables**

**Table S1.** **Baseline characteristics by red blood cell distribution width quartile**

|  | **1st quartile** | **2nd quartile** | **3rd quartile** | **4th quartile** | *P* |
| --- | --- | --- | --- | --- | --- |
|  | **<12.5%** | **<13.0% and ≥12.5%** | **<13.8% and ≥13.0%** | **≥13.8%** |  |
| Count | 3,817 | 3,947 | 4,351 | 4,302 |  |
| Age (years) | 52.5 [38.0, 64.0] | 55.2 [41.3, 66.0] | 57.0 [43.5, 68.0] | 57.0 [43.7, 68.4] | <0.001 |
| Sex |  |  |  |  | 0.19 |
| Female | 1,975 (51.7%) | 2,033 (51.5%) | 2,216 (50.9%) | 2,132 (49.6%) |  |
| Male | 1,842 (48.3%) | 1,914 (48.5%) | 2,135 (49.1%) | 2,170 (50.4%) |  |
| Body mass index (kg/m^2^) | 23.7 [21.2, 26.1] | 23.6 [21.3, 26.1] | 23.4 [21.1, 26.0] | 23.1 [20.6, 25.5] | <0.001 |
| Systolic blood pressure (mm Hg) | 130.0 [117.0, 144.0] | 130.0 [118.0, 146.0] | 130.0 [117.5, 146.0] | 130.0 [118.0, 147.0] | 0.217 |
| Diastolic blood pressure (mm Hg) | 79.0 [70.0, 88.0] | 79.0 [70.0, 88.0] | 79.0 [70.0, 88.0] | 79.0 [70.0, 88.0] | 0.74 |
| Hemoglobin (g/dL) | 13.4 [12.3, 14.7] | 13.1 [11.7, 14.5] | 12.5 [11.0, 14.0] | 10.8 [9.4, 12.4] | <0.001 |
| Serum creatinine (mg/dL) | 1.1 [0.8, 1.5] | 1.1 [0.9, 1.6] | 1.3 [0.9, 1.8] | 1.4 [1.0, 2.2] | <0.001 |
| Estimated glomerular filtration rate (mL/min/1.73 m^2^)^a^ | 67.8 [44.2, 93.5] | 61.9 [39.1, 90.0] | 54.7 [33.1, 81.9] | 46.4 [27.1, 72.8] | <0.001 |
| Urinary albumin grade |  |  |  |  | <0.001 |
| Negative | 2,071 (57.4%) | 2,030 (55.2%) | 2,132 (52.9%) | 1,872 (47.1%) |  |
| 1+ | 336 (9.3%) | 291 (7.9%) | 389 (9.7%) | 512 (12.9%) |  |
| 2+ | 544 (15.1%) | 669 (18.2%) | 708 (17.6%) | 808 (20.3%) |  |
| 3+ | 544 (15.1%) | 574 (15.6%) | 664 (16.5%) | 655 (16.5%) |  |
| 4+ | 110 (3.1%) | 114 (3.1%) | 136 (3.4%) | 126 (3.2%) |  |
| Serum albumin | 4.2 [4.0, 4.5] | 4.2 [3.9, 4.4] | 4.1 [3.8, 4.4] | 3.9 [3.5, 4.2] | <0.001 |
| History of hypertension | 1,092 (35.9%) | 1,213 (39.8%) | 1,477 (41.6%) | 1,554 (41.6%) | <0.001 |
| History of diabetes mellitus | 751 (26.1%) | 804 (28.9%) | 957 (29.6%) | 965 (28.6%) | 0.015 |
| Crude mortality rate | 301 (7.9%) | 348 (8.8%) | 511 (11.7%) | 646 (15.0%) | <0.001 |
| Crude end-stage renal disease incidence rate | 377 (9.9%) | 416 (10.5%) | 561 (12.9%) | 579 (13.5%) | <0.001 |

^a^Calculated using the Modification of Diet in Renal Disease equation.^1^

Values are presented as *n* (%) or as mean [interquartile range].

**Table S2.** **Baseline characteristics of participants by mortality group**

|  | **Alive** | **Late mortality (After 90days)** | **Early mortality (within 90days)** | **Excluded patients*** |
| --- | --- | --- | --- | --- |
| Count | 14611 (89.0) | 1088 (6.6) | 718 (4.4) | 2820 |
| High-RDW group (%) | 3656 (25.0) | 461 (42.4) | 185 (25.8) | 741 (26.3) |
| Baseline RDW (mean ± SD) | 13.3±1.5 | 13.9±1.7 | 13.4±1.6 | 13.4±1.7 |
| High time-averaged RDW group (%) | 3501 (24.0) | 480 (44.1) | 189 (26.3) | 719 (25.5) |
| Time-averaged RDW (mean ± SD) | 13.3±1.2 | 13.9±1.5 | 13.4±1.3 | 13.3±1.4 |
| Age (years) | 51.7±17.0 | 68.5±12.1 | 66.0±12.4 | 56.6±18.0 |
| Sex |  |  |  |  |
| Female | 7670 (52.5) | 440 (40.4) | 246 (34.4) | 1268 (45.5) |
| Male | 6941 (47.5) | 648 (59.6) | 472 (65.7) | 1552 (55.0) |
| Hemoglobin (g/dL) | 12.5±2.2 | 11.3±2.2 | 12.4±2.2 | 12.3±2.3 |
| Estimated glomerular filtration rate (mL/min/1.73 m^2^)^b^ | 64.6±39.2 | 48.6±30.0 | 51.3±30.4 | 60.4±40.4 |
| CKD grade |  |  |  |  |
| 1 | 3397 (23.2) | 108 (9.9) | 77 (10.7) | 578 (20.5) |
| 2 | 3775 (25.8) | 198 (18.2) | 181 (25.2) | 677 (24.0) |
| 3 | 4672 (32.0) | 469 (43.1) | 262 (36.5) | 892 (31.6) |
| 4 | 1521 (10.4) | 228 (21.0) | 115 (16.0) | 319 (11.3) |
| 5 | 1246 (8.5) | 85 (7.8) | 83 (11.6) | 353 (12.5) |
| Urinary albumin grade by urine analysis |  |  |  |  |
| Negative | 7250 (53.2) | 547 (53.8) | 318 (48.8) | 1267 (47.7) |
| 1+ | 1390 (10.2) | 44 (4.3) | 94 (14.4) | 360 (13.6) |
| 2+ | 2395 (17.6) | 239 (23.5) | 95 (14.6) | 454 (17.1) |
| 3+ | 2142 (15.7) | 178 (17.5) | 117 (17.9) | 367 (13.8) |
| 4+ | 449 (3.3) | 9 (0.9) | 28 (4.3) | 208 (7.8) |
| Serum albumin | 4.0±0.5 | 3.7±0.5 | 4.0±0.5 | 4.0±0.5 |
| History of hypertension | 4307 (29.5) | 663 (60.9) | 366 (51.0) | 812 (28.8) |
| History of diabetes mellitus | 2798 (19.1) | 396 (36.4) | 283 (39.4) | 615 (21.8) |
|  |  |  |  |  |

Excluded patients* that an observation period did not reach 30 days.

**Table S3.** **Baseline characteristics of the study population after propensity score matching.**

|  | Normal-RDW group^a^ | High-RDW group^a^ | *P* | *Standardized difference* |
| --- | --- | --- | --- | --- |
| Matched population | N= 3220 | N=3220 |  |  |
| Age (years) | 55.0 ± 17.2 | 55.0 ± 17.2 | 0.461 | 0.018 |
| Sex |  |  | 0.081 | 0.043 |
| Female | 1663 (51.6) | 1593 (49.5) |  |  |
| Male | 1557 (48.4) | 1627 (50.5) |  |  |
| Hemoglobin (g/dL) | 11.3 ± 1.8 | 11.0 ± 2.1 | <0.001 | -0.176 |
| Estimated glomerular filtration rate (mL/min/1.73 m^2^)^b^ | 55.7 ± 37.5 | 55.9 ± 37.7 | 0.773 | 0.007 |
| CKD grade |  |  | 0.270 |  |
| 1 | 549 (17.0) | 531 (16.5) |  |  |
| 2 | 657 (20.4) | 674 (20.9) |  |  |
| 3 | 1147 (35.6) | 1207 (37.5) |  |  |
| 4 | 498 (15.5) | 444 (13.8) |  |  |
| 5 | 369 (11.5) | 364 (11.3) |  |  |
| Urinary albumin grade by urine analysis |  |  | <0.001 | -0.046 |
| Negative | 1623 (50.4) | 1569 (48.7) |  |  |
| 1+ | 266 (8.3) | 414 (12.9) |  |  |
| 2+ | 606 (18.8) | 641 (19.9) |  |  |
| 3+ | 583 (18.1) | 474 (14.7) |  |  |
| 4+ | 142 (4.4) | 122 (3.8) |  |  |
| Serum albumin | 3.8 ± 0.5 | 3.7 ± 0.6 | <0.001 | -0.120 |
| History of hypertension | 1187 (36.9) | 1193 (37.1) | 0.857 | 0.004 |
| History of diabetes mellitus | 757 (22.9) | 730 (22.7) | 0.835 | -0.005 |
| RDW at baseline | 12.7 ± 0.5 | 15.4 ± 1.9 | <0.001 |  |
| Time averaged RDW | 13.0 ± 0.8 | 14.7 ± 1.5 | <0.001 |  |

**Table S4.** Hazard ratios of all-cause mortality according to RDW group after propensity score matching

|  | Model A | | Model B | | Model C | |
| --- | --- | --- | --- | --- | --- | --- |
|  | Adjusted HR^a^  (95% CI) | *P* | Adjusted HR^b^  (95% CI) | *P* | Adjusted HR^c^  (95% CI) | *P* |
| High-RDW group (vs. normal-RDW group) | 1.389 (1.210–1.594) | <0.001 | 1.347 (1.172–1.549) | <0.001 | **1.329 (1.155–1.529)** | **<0.001** |
| Baseline RDW (as a continuous variable, per 1-unit increase) | 1.094 (1.058–1.132) | <0.001 | 1.088 (1.052–1.126) | <0.001 | **1.083 (1.047–1.122)** | **<0.001** |
| High time-averaged RDW group (vs. normal time-averaged group)^d^ | 1.619 (1.408–1.861) | <0.001 | 1.543 (1.341–1.777) | <0.001 | **1.530 (1.326–1.765)** | **<0.001** |
| Time-averaged RDW (as a continuous variable, per 1-unit increase)^d^ | 1.201 (1.154–1.251) | <0.001 | 1.190 (1.142–1.240) | <0.001 | **1.186 (1.137–1.237)** | **<0.001** |

^a^Model A was adjusted for age, sex, estimated glomerular filtration rate, and baseline hemoglobin level.

^b^Model B was adjusted for all covariates in model A plus history of diabetes mellitus, history of hypertension, serum albumin level, and urinary albumin grade.

^c^Model C was adjusted for all covariates in model B plus history of red blood cell transfusion and use of erythropoietin-stimulating agents.

^d^When analysis was performed with time-averaged RDW values, the time-averaged hemoglobin levels and time-averaged estimated glomerular filtration rate were included in the multivariate Cox regression model.

Abbreviations: CI, confidence interval; HR, hazard ratio; RDW, red blood cell distribution width

**References**

1. Levey AS, Bosch JP, Lewis JB, Greene T, Rogers N, Roth D. A more accurate method to estimate glomerular filtration rate from serum creatinine: a new prediction equation. Modification of Diet in Renal Disease Study Group. Ann Intern Med. 1999;130(6):461-470.

The Korean Association for the study of Renal Anemia and artificial Intelligence (KARAI)*

Chun Soo Lim, Seoul National University College of Medicine, Seoul, Korea

President

Sung Gyun Kim, Hallym University College of Medicine, Anyang, Korea

Vice-President

Gang Jee Ko, Korea University College of Medicine, Seoul, Korea

Secretary General

Jung Tak Park, Yonsei University College of Medicine, Seoul, Korea

Director, the Scientific Programs

Tae Ik Chang, National Health Insurance Service Ilsan Hospital, Goyang, Korea

Director, the Public Relation

Sungjin Chung, College of Medicine, The Catholic University of Korea, Seoul, Korea

Director, the Planning

Jung Pyo Lee, Seoul National University College of Medicine, Seoul, Korea

Director, the Publication

Dong Ki Kim, Seoul National University College of Medicine, Seoul, Korea

Treasurer

Sang Ho Lee, College of Medicine, Kyung Hee University, Seoul, Korea

Director, at Large

Bum Soon Choi, College of Medicine, The Catholic University of Korea, Seoul, Korea

Director, at Large

Jin Seok Jeon, Soon Chun Hyang University, Seoul, Korea

Director, at Large

Sangheon Song, Pusan National University College of Medicine, Busan, Korea

Director, at Large

Dae Eun Choi, Chungnam National University College of Medicine, Daejeon, Korea

Director, at Large

Dong Ryeol Ryu, Yonsei Jungsung Clinic, Seoul, Korea

Director, at Large

Woo Kyung Jung, Gachon University of Medicine and Science, Incheon, Korea

Auditor

( available at <https://karai.or.kr/content/staff> 19 October 2021, This is the current list of executive committees.)
