# Supplementary material for: Red blood cell distribution width as a predictor of mortality among patients regularly visiting the nephrology outpatient clinic
Source: Sci Rep. 2021 Dec 21;11:24310. doi: 10.1038/s41598-021-03530-2 (PMC8692533; doi:10.1038/s41598-021-03530-2)
Supplement: Supplementary file 2 — Supplementary Figures. [file 41598_2021_3530_MOESM2_ESM.pdf]

Figure S1.

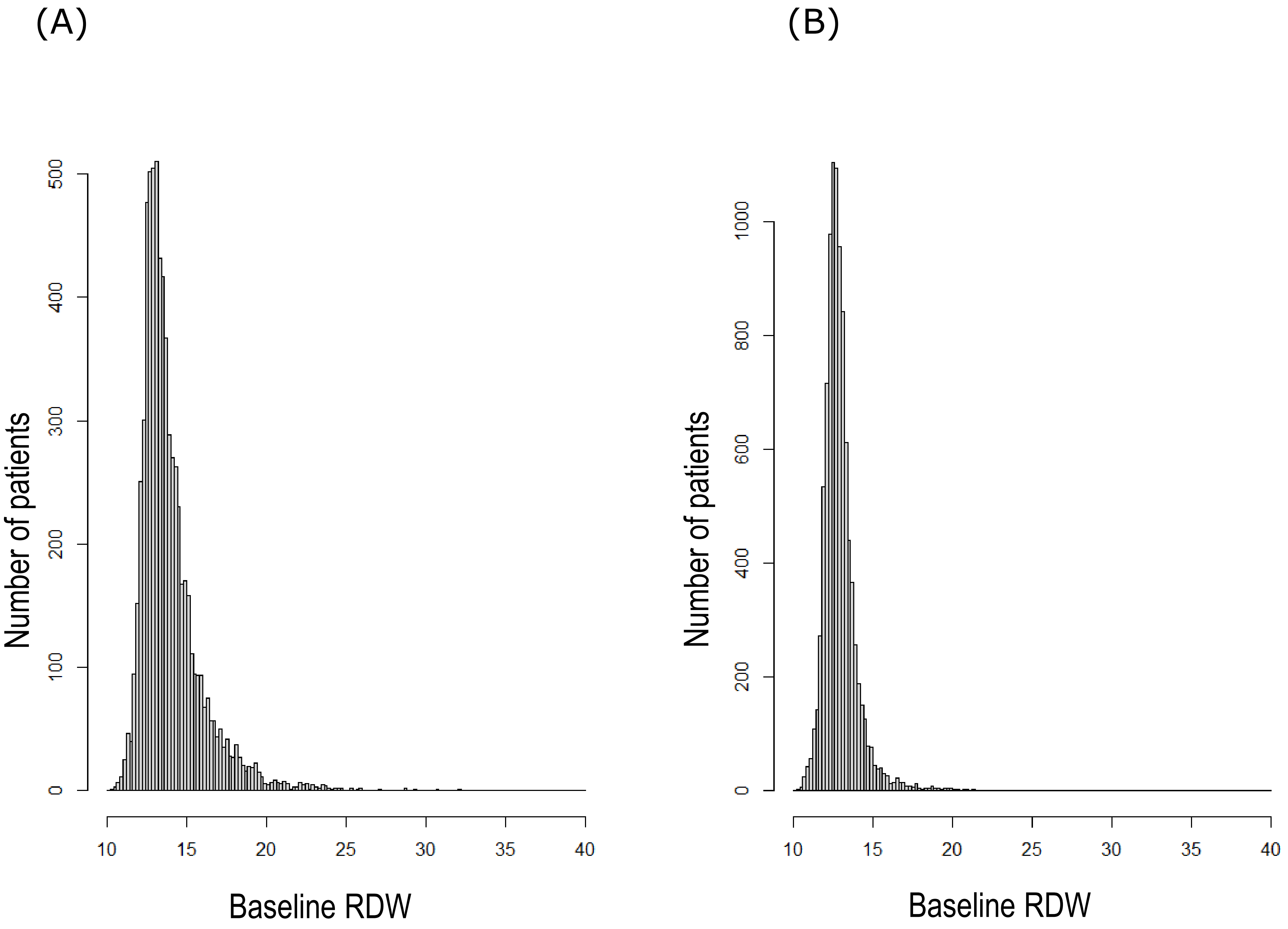
(A)
(B)
Number of patients
Baseline RDW
0
100
200
300
400
500
10
15
20
25
30
35
40
0
200
400
600
800
1000

Figure S2.

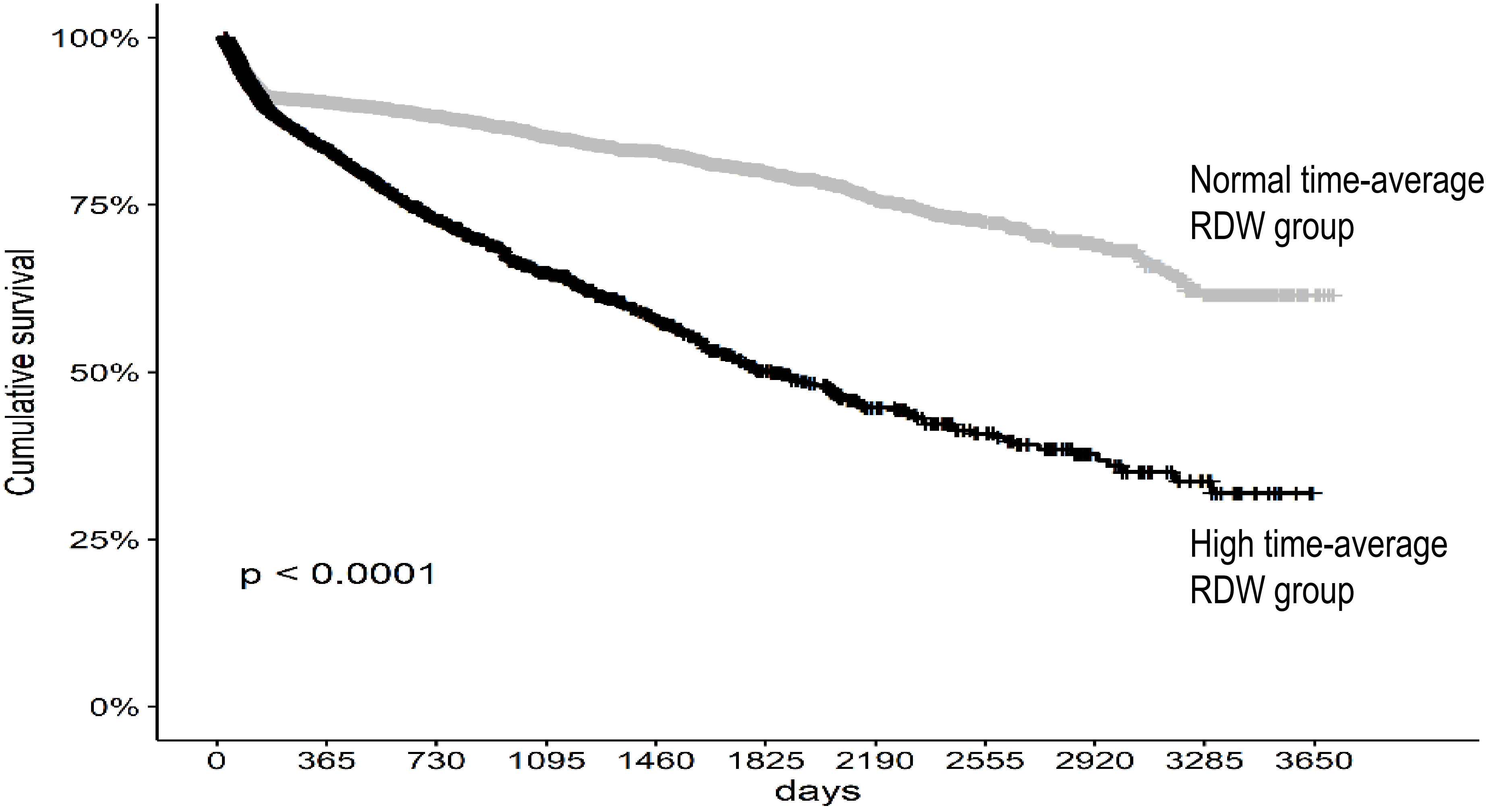

	0	365	730	1095	1460	1825	2190	2555	2920	3285	3650
Normal time-average RDW group	12247	3027	2362	1788	1338	965	613	414	252	89	5
High time-average RDW group	4170	932	653	456	312	202	122	79	43	21	0

days

Figure S3.

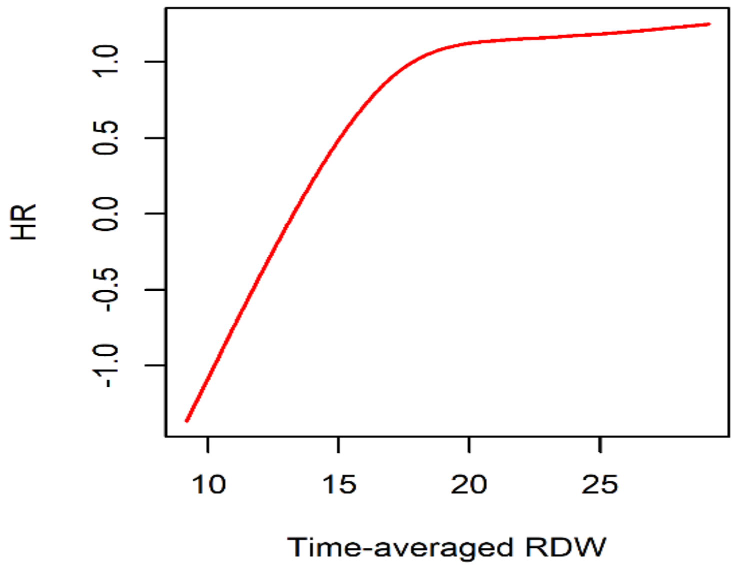
HR
-1.0
-0.5
0.0
0.5
1.0
10
15
20
25
Time-averaged RDW

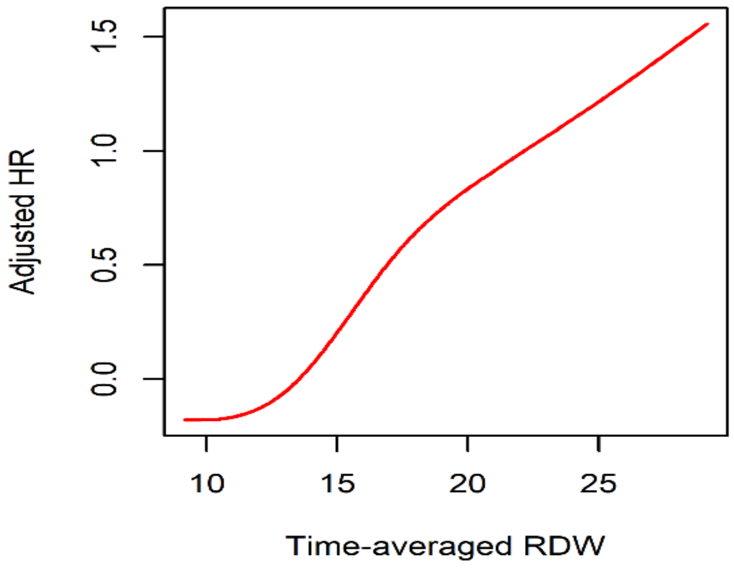
Adjusted HR
0.0
0.5
1.0
1.5
10
15
20
25
Time-averaged RDW

Figure S4.

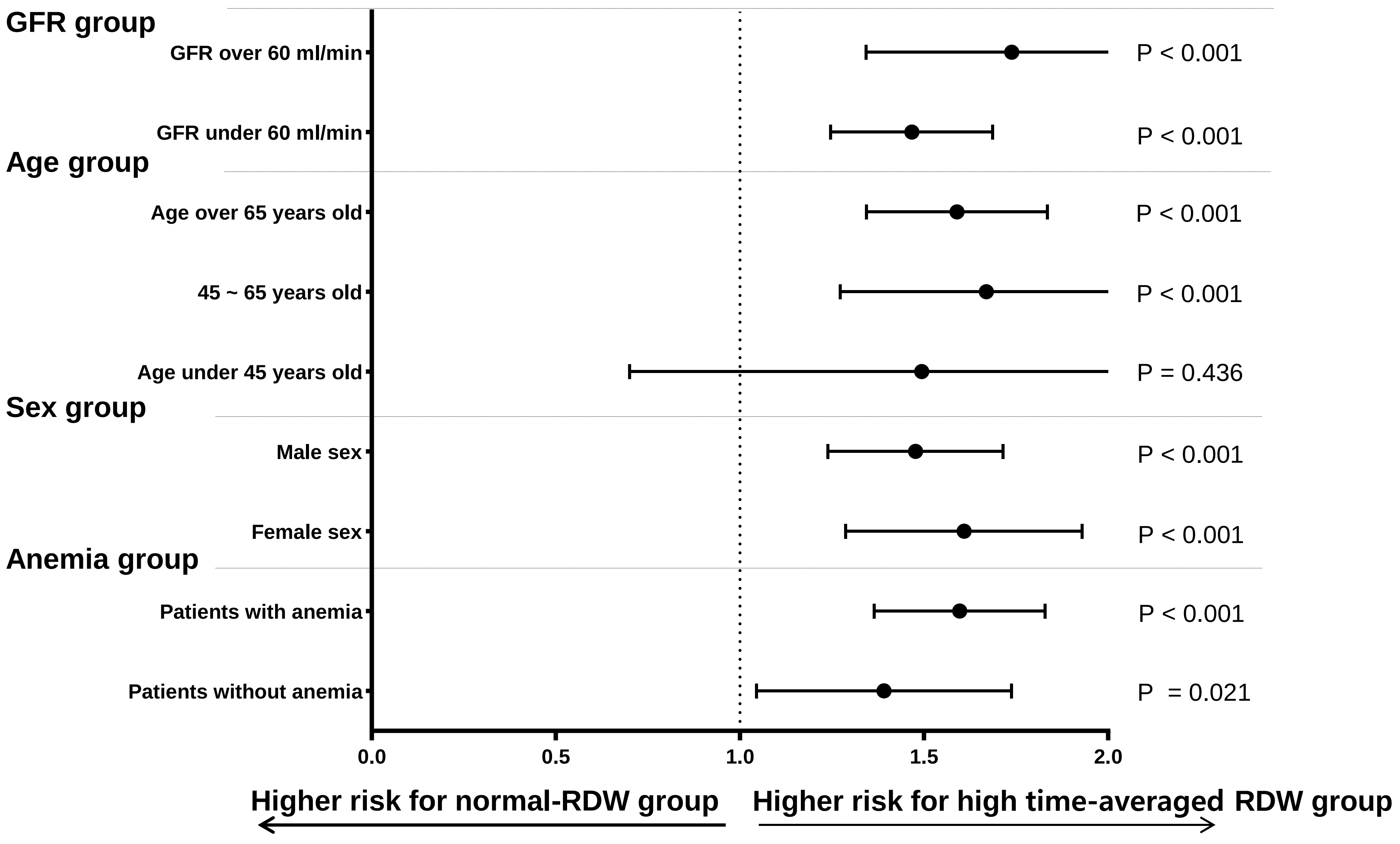
GFR group
GFR over 60 ml/min
P < 0.001
GFR under 60 ml/min
P < 0.001
Age group
Age over 65 years old
P < 0.001
45 ~ 65 years old
P < 0.001
Age under 45 years old
P = 0.436
Sex group
Male sex
P < 0.001
Female sex
P < 0.001
Anemia group
Patients with anemia
P < 0.001
Patients without anemia
P = 0.021
0.0
0.5
1.0
1.5
2.0
Higher risk for normal-RDW group
Higher risk for high time-averaged RDW group

Figure S5.

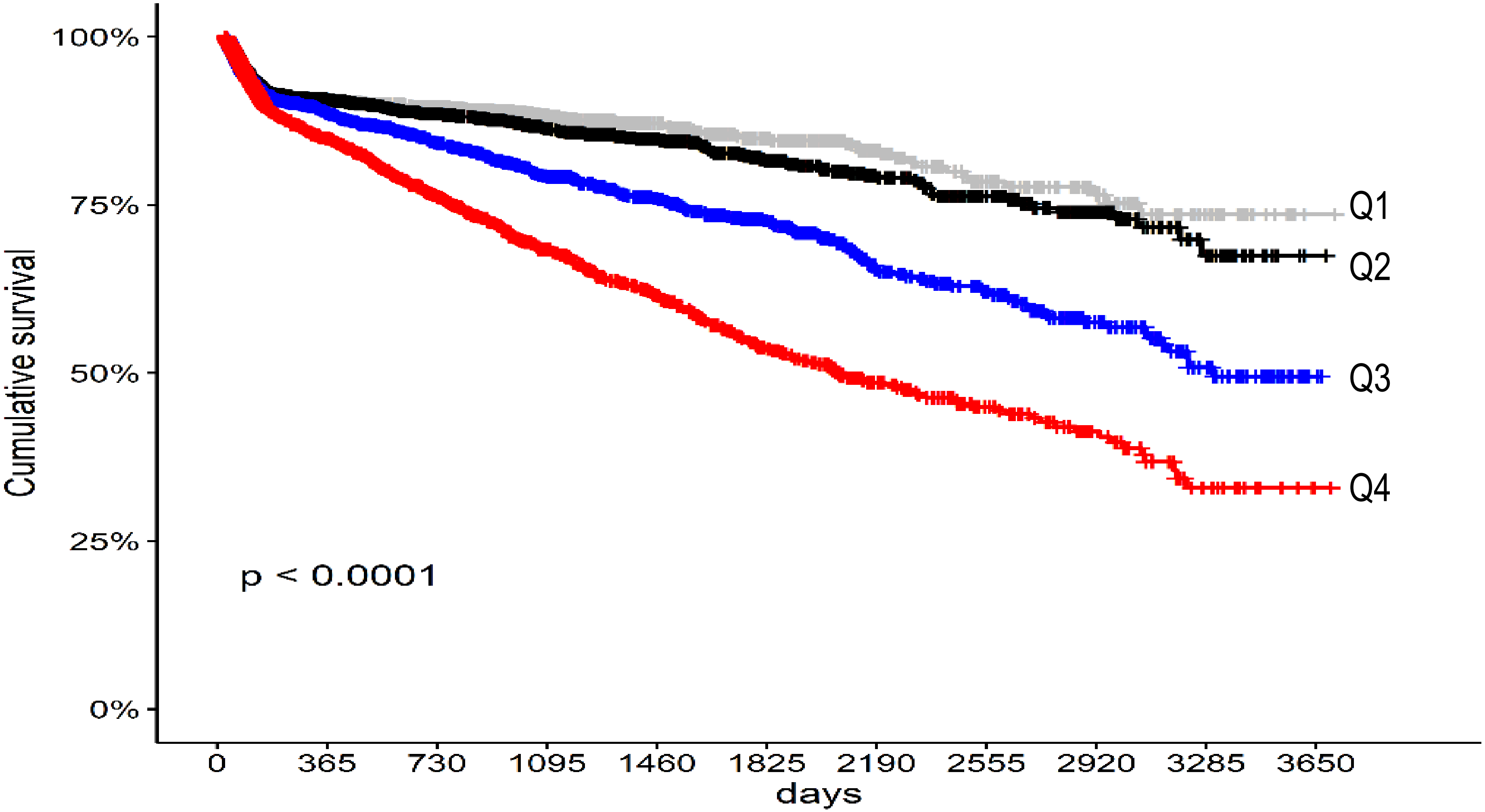

No. at risk

	0	365	730	1095	1460	1825	2190	2555	2920	3285	3650
Q1	3817	805	642	486	350	250	154	100	64	22	1
Q2	3947	1060	816	621	480	352	234	158	93	28	1
Q3	4351	1136	861	647	480	339	204	145	86	40	2
Q4	4302	958	696	490	340	226	143	90	52	20	1

days

Figure S6.

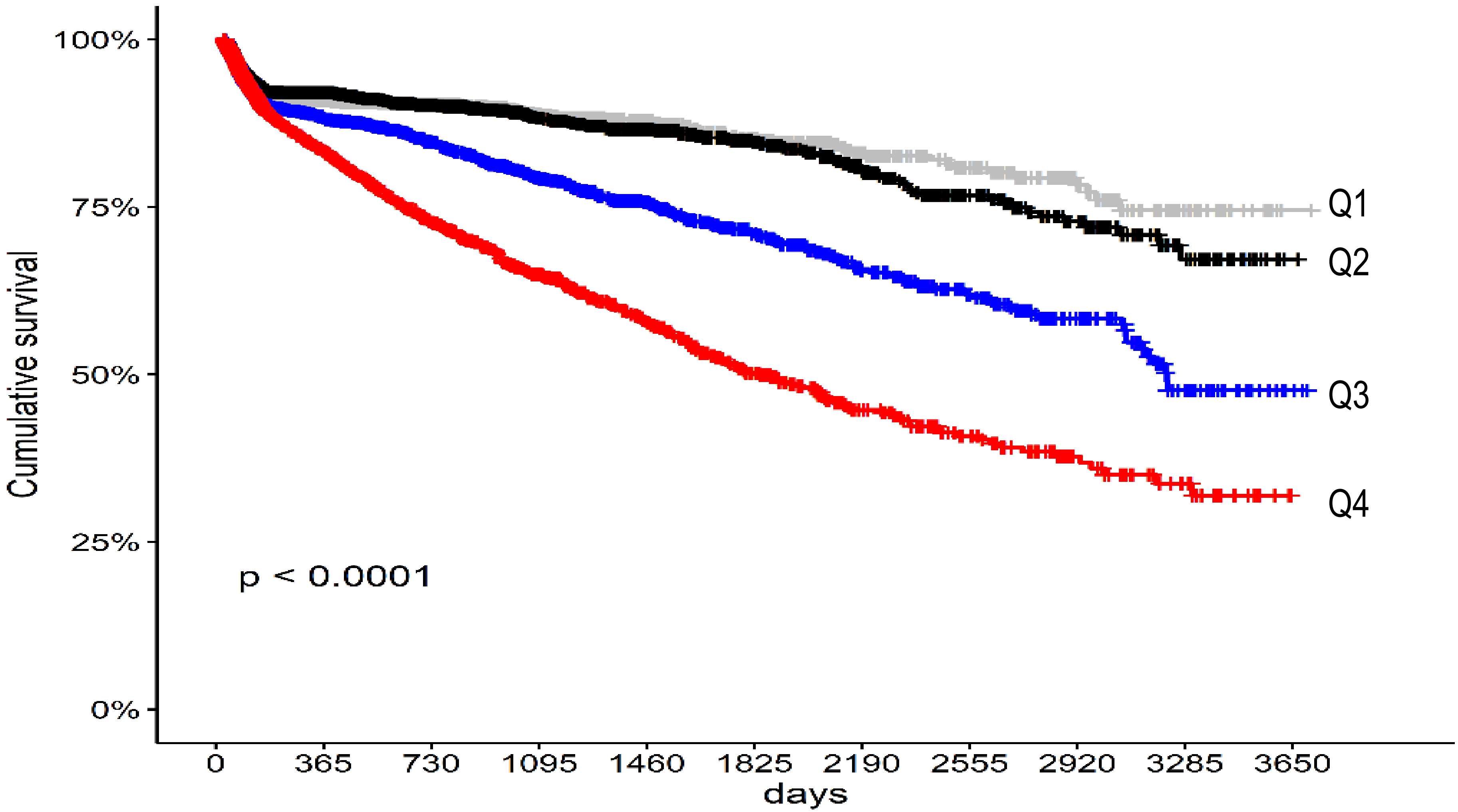

No. at risk

	0	365	730	1095	1460	1825	2190	2555	2920	3285	3650
Q1	4131	912	725	564	415	300	187	128	73	24	1
Q2	3980	1050	825	621	469	352	223	145	94	32	1
Q3	4136	1065	812	603	454	313	203	141	85	33	3
Q4	4170	932	653	456	312	202	122	79	43	21	0

days

Figure S7.

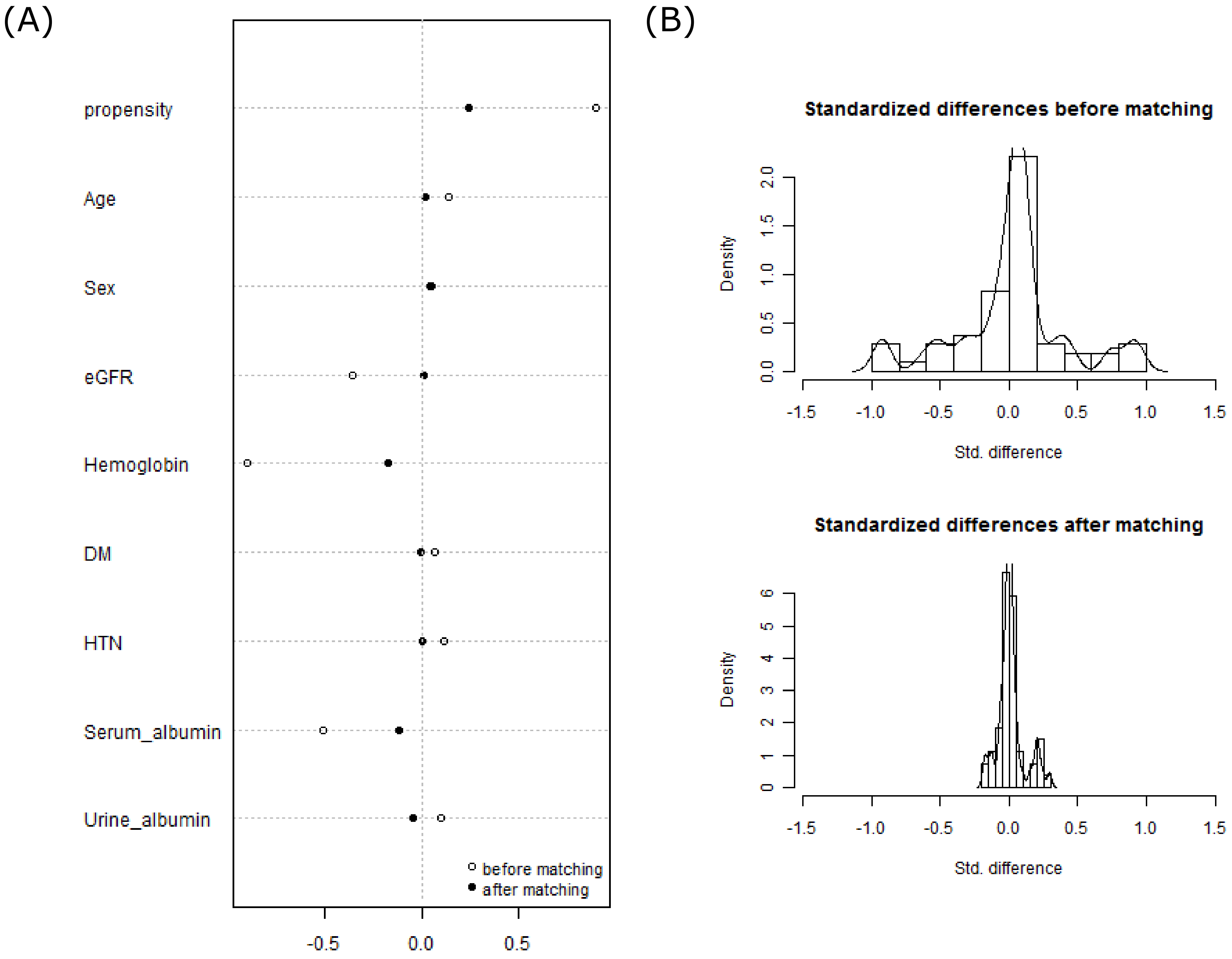
(A)
propensity
Age
Sex
eGFR
Hemoglobin
DM
HTN
Serum_albumin
Urine_albumin
before matching
after matching
-0.5
0.0
0.5
(B)
Standardized differences before matching
Density
0.0
0.5
1.0
1.5
2.0
-1.5
-1.0
-0.5
0.0
0.5
1.0
1.5
Std. difference
Standardized differences after matching
Density
0
1
2
3
4
5
6
-1.5
-1.0
-0.5
0.0
0.5
1.0
1.5
Std. difference
